# Injuries in the North – analysis of 20 years of surveillance data collected by the Canadian Hospitals Injury Reporting and Prevention Program

**DOI:** 10.3402/ijch.v72i0.21090

**Published:** 2013-09-20

**Authors:** Minh T. Do, Mylène Fréchette, Steven McFaull, Bryany Denning, Mike Ruta, Wendy Thompson

**Affiliations:** 1 Injury Section, Health Surveillance and Epidemiology Division, Public Health Agency of Canada, Ottawa, ON, Canada; 2 Department of Health and Social Services, Northwest Territories, Canada; 3 Health and Social Services, Nunavut, Canada

**Keywords:** injury, CHIRPP, Nunavut, Northwest Territories, surveillance

## Abstract

**Background:**

Injury is a major public health concern, particularly for Canadians living in Arctic regions where the harsh physical and social conditions pose additional challenges. Surveillance data collected over the past 2 decades through the Canadian Hospitals Injury Reporting and Prevention Program (CHIRPP) provide insights into the burden of injuries in certain parts of Canada.

**Objectives:**

This study aims to summarize and compare patterns of injuries in the Northwest Territories (NWT) and Nunavut to other southern communities across Canada.

**Methods:**

Analysis was based on CHIRPP data covering the period 1991–2010. Proportionate injury ratio (PIR) and its 95% confidence interval were used to summarize and compare the injury experience of Canadians living in the Arctic regions to other CHIRPP sites across Canada.

**Results:**

Between 1991 and 2010, there were 65,116 reported injuries. Approximately 83% of the cases were unintentional in nature; however, significantly higher proportions were observed for assaults and maltreatment (PIR=2.80, 95% CI: 2.72–2.88) among Canadians living in northern communities. Significantly higher proportions were also observed for crushing/amputations (PIR=2.28, 95% CI: 2.14–2.44), poison/toxic effects (PIR=1.21, 95% CI: 1.15–1.28), drowning/asphyxiations (PIR=1.52, 95% CI: 1.33–1.74) and frostbites (PIR=7.39, 95% CI: 6.60–8.28). The use of all-terrain vehicles or snowmobiles also resulted in significantly higher proportions of injuries (PIR=1.93, 95% CI: 1.79–2.09).

**Conclusions:**

This study contributes to the limited literature describing injuries in northern communities where the harsh physical and social climates pose additional challenges. Excesses in the proportions identified in this study could be useful in identifying strategies needed to minimize injury risks in northern communities within Canada.

Although most injuries are predictable and preventable, clinicians of emergency departments across Canada are inundated with injured patients needing medical attention on a daily basis. Injuries have a major impact on the quality of life and also represent a significant burden to the health care system. To date, injury remains the leading cause of death among Canadians between 1 and 44 years of age (1), accounting for 190,000 years of life lost (2) and costing approximately $20 billion annually (3).

The seriousness of the problem is magnified among populations living in the northern communities where residents face extreme weather conditions, physical geographic remoteness and acute social issues. In the Northwest Territories (NWT) and Nunavut, many communities are only accessible by air during all or part of the year (4). Also, only a few major population centres have hospitals, with the majority of communities relying on health centres staffed by nurses or nurse practitioners. These health centres have limited diagnostic equipment and treatment capacity. As a result, individuals with serious injuries in many communities need to be evacuated by air to the nearest major centre, an expensive process which can take many hours and be delayed indefinitely by inclement weather (5). In the case of injuries due to interpersonal violence, or self-inflicted injuries, the social environment also poses challenges, as many communities have limited or no mental health or family violence counselling resources or family violence shelters, and some communities have no routine services from the Royal Canadian Mounted Police or local law enforcement, other than on an emergency or fly-in basis (6–8). The social, structural and geographic conditions in the NWT and Nunavut both increase the risk of injury, and complicate the treatment of injury when it occurs.

To enhance our understanding of the mechanism of injuries, the Public Health Agency of Canada conducts injury surveillance through the Canadian Hospitals Injury Reporting and Prevention Program (CHIRPP) (9). For the past 20 years, CHIRPP has been collecting data from different geographic areas including those of the NWT and Nunavut. The CHIRPP is an injury and poisoning surveillance system currently operating in 11 paediatric and 5 general hospitals beginning in Canada in 1991. The CHIRPP system currently contains over 2.2 million records containing information about activity at the time of injury, the direct cause of the injury, contributing factors, time and place of the injury event, the patients’ age and sex, up to 3 injuries (body part and nature of injury) and the treatment received in the emergency department. Narrative fields provide information to further refine the coding and identify rare events. Although only selected hospitals report to CHIRPP, previous work has shown that the data collected through the programme represent general injury patterns among Canadian Youth (10). The purpose of this study is to report on CHIRPP injury data collected from 2 territories of Canada over the past 2 decades. Specifically, this study aims to describe patterns of injury in order to better understand the burden of injuries in the North and to identify opportunities for improvement.

## Method

### Data source and handling

Analyses were based on data collected by the CHIRPP. CHIRPP is an emergency department-based injury surveillance system operating in different health centres across Canada since 1991. Within the NWT and Nunavut, CHIRPP collected injury data from Stanton Regional Hospital as well as Fort Smith Health, Inuvik, Gjoa Haven, Baffin Nurse station (in Iqaluit) and Arctic Bay health centres. For each recorded injury, demographic information as well as time, place, nature, intent and activity at the time of injury were also extracted for analysis. Given that some individuals may have multiple injuries in a given event, the unit of analysis in this study is the injury event. All data are coded consistently across CHIRPP sites. Coding of injury data in CHIRPP has been described elsewhere and data from CHIRPP have been validated and are considered good quality (9–16).

### Statistical analysis

Injury records with missing information on sex (n=28) and error in date of registration (n=2) were excluded. Proportionate injury ratios (PIRs) were used to compare injuries of northern communities to age-specific proportions (5-year age groups) of other CHIRPP sites across Canada (17). The 95% confidence interval (α=0.05) is the criterion used to determine statistical significance and was computed using methodology described by Breslow and Day (18). A PIR of 1 indicates that the observed proportion of cases for a given injury is the same as the sum of the age-specific proportions of that injury. For example, a PIR of 1.5 represents 50% more than expected, while a PIR of 0.5 indicates 50% less than expected. Analyses were conducted using SAS Version 9.2 (19) and Microsoft Excel tools.

## Results

**Fig. 1:**
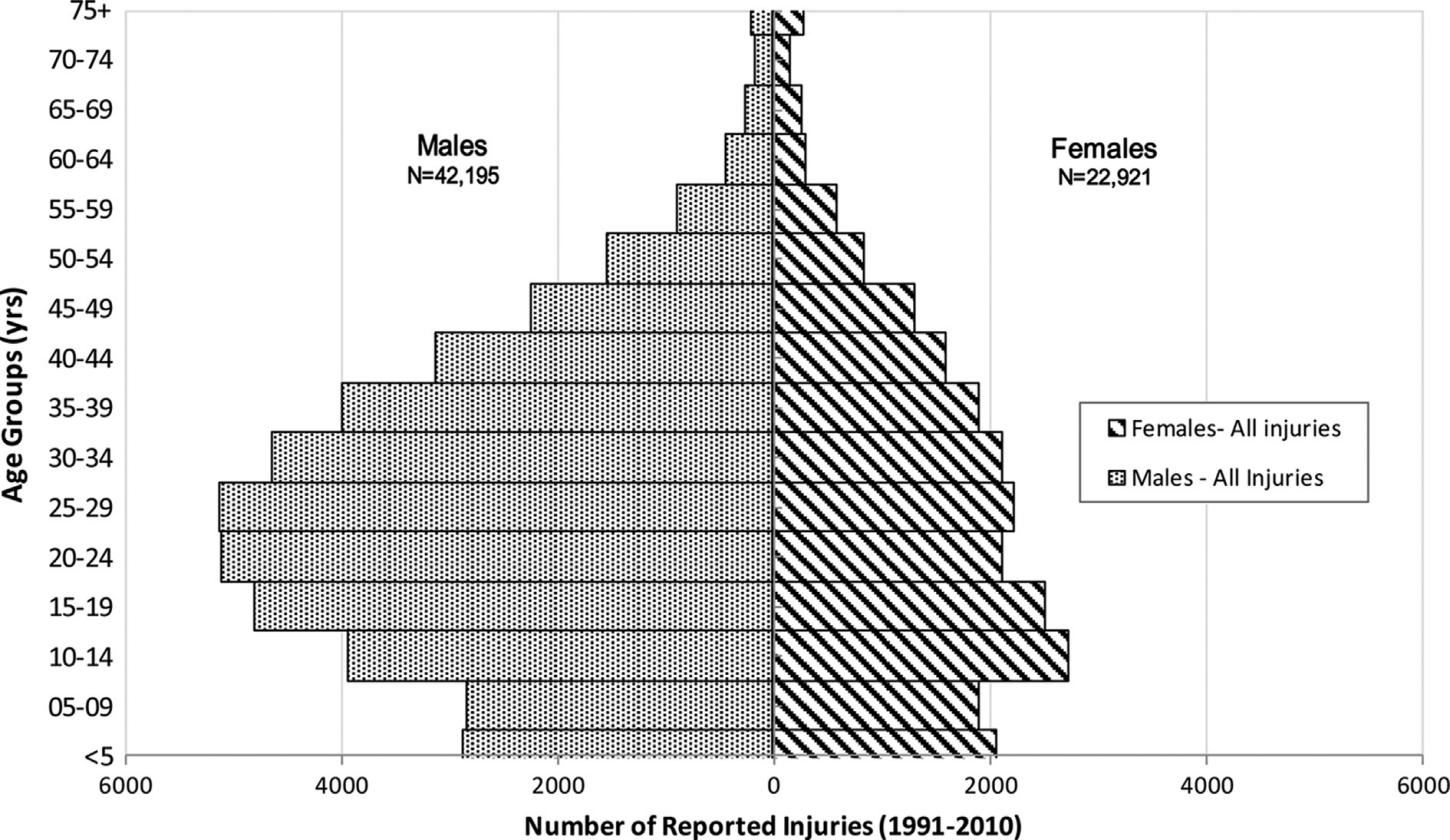
Age and gender distributions of injuries reported to the Canadian Hospitals Injury Reporting and Prevention Program (CHIRPP) from communities within the Northwest Territories (NWT) and Nunavut, 1991–2010.

Between 1991 and 2010, 65,116 injuries were reported to CHIRPP from 8 health care facilities located within the NWT and Nunavut. Figure 1 shows the age distribution of injury by sex. Overall, males sustained almost twice the number of reported injuries (n=42,195) as compared to females (n=22,921) with most of the injuries occurring between the ages of 10 and 44 years.

Of the injuries reported to CHIRPP (Table I), more than 90% of the cases were from the NWT. This was expected given that the largest territorial data collection sites and the only hospital were located in NWT. Of all the injuries reported, 84% were unintentional in nature. However, more than 4,000 incidents of assaults and intentional self-harm were also reported. While almost half of the injuries were relatively minor, some were more serious, involving fractures (11%), brain injuries (3.5%) and crushing/amputation (1.4%). Injuries resulting in poisoning (2%) and drowning/near drowning or asphyxiations (0.3%) were also reported.

**Table I:** Distribution of injury characteristics reported to the Canadian Hospitals Injury Reporting and Prevention Program (CHIRPP) from communities within the Northwest Territories (NWT) and Nunavut, 1991–2010

	Numberα of injuries by gender		
	
Injury characteristics	Males	Females	Totalβ (%)
Intent of injuryβ			
Unintentional	35,511	19,035	54,546 (83.8)
Intentional self-harm	415	511	926 (1.4)
Assault and maltreatment*	3,161	1,619	4,780 (7.3)
Other not specified	3,108	1,756	4,864 (7.5)
Nature of Injuryβ			
Superficial/open wound/soft tissue	21,030	10,230	31,260 (48.0)
Dislocation/sprain/strain/pulled elbow	8,176	5,462	13,638 (20.9)
Fractures	4,469	2,472	6,941 (10.7)
Brain injury	1,456	812	2,268 (3.5)
Foreign body	1,550	582	2,132 (3.3)
Poison/toxic effects	664	724	1,388 (2.1)
Eye/globe	997	372	1,369 (2.1)
Injury to nerve/vessel/tendon	854	487	1,341 (2.1)
Burns/corrosion	769	514	1,283 (2.0)
Crushing/amputation	676	229	905 (1.4)
Frostbites	217	82	299 (0.5)
Drowning and other asphyxias	134	82	216 (0.3)
Activity at the time of injuryβ			
Occupational	8,827	1,732	10,559 (16.2)
Maintenance and chores	4,176	1,962	6,138 (9.4)
Other sports	2,856	1,307	4,163 (6.4)
Leisure activities	1,975	1,046	3,021 (4.6)
Organized sports	1,983	748	2,731 (4.2)
Other transports	930	828	1,758 (2.7)
Vital activities	679	652	1,331 (2.0)
Bicycling	864	334	1,198 (1.8)
Cooking and food preparations	395	356	751 (1.1)
ATV and snowmobiles	459	177	636 (1.0)
Fighting	362	89	451 (0.7)
Pedestrian	173	130	303 (0.5)
Number of injuries admitted and/or transferred to a hospital			
Admitted or transferred to a hospital	2,303	1,427	3,730 (5.7)

α One CHIRPP record is completed for every injury event;

β excluding unknown/missing; ATV, all-terrain vehicles.

* Assaults (sexual, unspecified, victims of assault) and maltreatment (by parent/caregiver or spouse/partner).

The distribution of injuries and the PIRs for the intent, nature, activity at the time of injury and admission status due to the injury are presented in Table II. Based on the intent of the injury, the PIR for assault and maltreatment in the North was disproportionately larger than all of the other CHIRPP sites across Canada combined, particularly for women (PIR=3.57, 95% CI: 3.40–3.75). Among the nature of injuries examined, significantly elevated proportions were observed in the North for the more serious injuries, namely poisonings, burns, crushing/amputations, and drowning/near drowning or other asphyxiations. Frostbite was also highly significant; however, this was based on relatively few cases. Based on the distribution of the activities engaged in at the time of the injury, the PIRs for organized sports, use of all-terrain vehicles (ATVs) and snowmobiles, and pedestrian-related activities were significantly higher in the North. When compared to the other CHIRPP sites across Canada, the proportion of injuries in the North being admitted or transferred to a hospital was significantly lower.

**Table II:** Proportionate injury ratio (PIR) (17) and confidence interval (95% CI) (18) by gender and selected injury characteristics reported to the Canadian Hospitals Injury Reporting and Prevention Program (CHIRPP) from communities within the Northwest Territories (NWT) and Nunavut, 1991–2010

	Total			Males			Females		
			
Injury characteristics	PIR	(95% CI)		PIR	(95% CI)		PIR	(95% CI)	
Intent of injury									
Unintentional	0.93	0.92	0.94	0.93	0.92	0.94	0.93	0.91	0.94
Intentional self-harm	0.94	0.88	1.00	1.03	0.93	1.13	0.93	0.85	1.01
Assault and maltreatment*	2.80	2.72	2.88	2.47	2.39	2.56	3.57	3.40	3.75
Nature of injury									
Superficial/open wound/soft tissue	1.07	1.06	1.08	1.86	1.84	1.88	1.02	1.00	1.04
Dislocation/sprain/strain/pulled elbow	1.37	1.34	1.39	1.36	1.33	1.39	1.40	1.36	1.44
Fractures	0.62	0.60	0.63	0.61	0.59	0.63	0.63	0.61	0.66
Brain injury	0.76	0.73	0.79	0.77	0.73	0.81	0.75	0.70	0.80
Foreign body	1.00	0.96	1.05	0.92	0.88	0.97	1.16	1.07	1.25
Poison/toxic effects	1.21	1.15	1.28	1.41	1.31	1.52	1.14	1.06	1.22
Eye (globe)	0.79	0.75	0.84	0.78	0.73	0.83	0.80	0.72	0.88
Injury to nerve/vessel/tendon	0.72	0.68	0.76	0.67	0.62	0.71	0.83	0.76	0.91
Burns/corrosion	1.33	1.26	1.40	1.35	1.26	1.45	1.34	1.23	1.46
Crushing/amputation	2.28	2.14	2.44	2.20	2.04	2.37	2.30	2.02	2.62
Frostbites	7.39	6.60	8.28	6.92	6.06	7.91	15.67	12.62	19.46
Drowning and other asphyxias	1.52	1.33	1.74	1.50	1.26	1.77	1.58	1.27	1.96
Activity at the time of injury									
Occupational	1.19	1.17	1.22	1.26	1.23	1.29	0.85	0.81	0.89
Maintenance and chores	1.06	1.03	1.08	0.94	0.91	0.97	1.31	1.25	1.37
Other sports	0.68	0.66	0.70	0.64	0.62	0.66	0.77	0.73	0.81
Leisure activities	1.23	1.19	1.27	1.29	1.23	1.35	1.14	1.07	1.21
Organized sports	1.93	1.79	2.09	1.65	1.51	1.81	2.85	2.46	3.30
Other transports	0.50	0.47	0.52	0.48	0.45	0.51	0.54	0.51	0.58
Vital activities	1.12	1.06	1.18	1.12	1.04	1.21	1.17	1.08	1.26
Bicycling	0.81	0.76	0.85	0.77	0.72	0.83	0.87	0.78	0.96
Cooking and food preparations	1.08	1.01	1.16	1.23	1.12	1.36	1.02	0.92	1.13
ATV and snowmobiles	1.93	1.79	2.09	1.65	1.51	1.81	2.85	2.46	3.30
Fighting	1.48	1.35	1.62	1.40	1.27	1.56	1.60	1.30	1.97
Pedestrian	1.93	1.79	2.09	1.65	1.51	1.81	2.85	2.46	3.30
Number of injuries admitted and/or transferred to a hospital									
Admitted or transferred to a hospital	0.71	0.69	0.74	0.63	0.60	0.66	0.88	0.84	0.93

* Assaults (sexual, unspecified, victims of assault) and maltreatment (by parent/caregiver or spouse/partner).

ATV, all-terrain vehicles.

## Discussions

In this study, we described the cause-specific injuries (1991–2010) of Canadians living in the North. Based on the analysis of 20 years of surveillance data collected by CHIRPP, our findings reaffirm the importance of assaults and maltreatment (PIR=2.80) among Canadians living in northern communities as previously reported elsewhere (20).

Consistent with other reports, injuries relating to ATV and snowmobiling are reported in significantly higher proportion than in the south (PIR=1.93, 95% CI: 1.79–2.09). In southern Canada, ATV and snowmobiles are often used for recreational purposes. However, in many northern and remote communities, these are used as a main method of transportation. In the NWT alone, ATV and snowmobiling account for 5% of all injury-related hospitalizations (20). Hospitalizations for ATV and snowmobiling-related injuries are much higher in the Territories than that of Canada combined (21).

Our analysis of CHIRPP data also showed gender differences in ATV and snowmobiling-related injuries. Within the North, there were fewer women injured (n=177) compared to males (n=459) likely reflecting more prevalent male users. However, when comparing with CHIRPP injuries elsewhere in Canada, ATV and snowmobiling-related injuries occurred much more frequently in females in the North (PIR=2.85, 95% CI=2.46–3.30) than that in males (PIR=1.65, 95% CI=1.51–1.81). Similar findings have been reported in the literature (22, 23). Further investigation is being conducted to see if the use of personal protective and anthropometric measures modifies the ATV and snowmobiling-related injury risks for males and females.

As expected, given the extreme climatic conditions, the results demonstrate highly significant excesses in the proportion of reported cases of frostbites from northern communities. Although the epidemiology of frostbites in Canada is not well understood, Urschel's review of 79 cases over a 10-year period from a northern Canadian hospital found that 53% of patients were under the influence of alcohol and 16% were suffering from psychiatric illnesses (24). In other Arctic countries, frostbites have been recognized as an important public health issue. In Finland, the incidence of frostbite was estimated to be approximately 2.5 per 100,000 residents (25). While the number of reported frostbite cases in the North was relatively small (n=299 over a 20-year period), the PIR is significantly higher than that of other CHIRPP sites. Frostbite injury is preventable, and therefore further work is being conducted to better understand the causes and circumstances so that more effective prevention measures may be put in place.

While our analysis is based on a large data set collected over a 20-year period, there are limitations that should be considered in interpreting the results. Our analysis was based on reported cases within CHIRPP sites and therefore, the numbers presented herein underestimate the true burden of injuries in the North. The extent of under-reporting needs to be quantified, but it is beyond the scope of this report. Although our data were collected from 7 CHIRPP sites made up of 1 large hospital and smaller health centres from different geographical areas within the Territories, it is not population based and the results are not generalizable to all communities within the North.

In our analysis, we used all provincial CHIRPP sites including the paediatric hospitals in order to have a stable standard for comparison. Given that the PIRs for injuries in the North included all age groups, sensitivity analyses were conducted to determine the potential impact of including paediatric hospitals in the calculation. Our results showed little difference as the confidence intervals overlapped. For example, the PIR for occupational-related activities, when excluding the paediatric hospitals, changed from 1.19 (95% CI: 1.17–1.22) to 1.14 (95% CI: 1.12–1.16).

In our analysis, we used the PIR as a statistical tool to compare the distribution of injuries in the North to the other CHIRPP sites across Canada. As reported by others, this statistic has 2 limitations, namely: (a) injury risks cannot be attributed to exposures and (b) the sum of all proportions must equal to unity (26). Despite these limitations, PIR are frequently used in other areas of research as they are useful in identifying potential injury risks (27). Excess in proportions, adjusted for potential confounders such as age and sex can be a valuable source of information for targeted prevention activities and further research.

## Conclusion

Injuries are important causes of morbidity and mortality, and in the North, the seriousness of the problem is magnified as a result of the additional challenges posed by geographic, environmental and social environments. This study highlights some of the causes of disproportionate injuries in the North. In particular, the NWT and Nunavut experience disproportionate distributions of intentional injuries such as assaults and maltreatment, as well as unintentional injuries relating to ATV use snowmobiling and exposure to severe cold.
